# Comparison of injury severity scores (ISS) obtained by manual coding versus “Two-step conversion” from ICD-9-CM

**DOI:** 10.1371/journal.pone.0216206

**Published:** 2019-05-01

**Authors:** Rebeca Abajas-Bustillo, Francisco José Amo-Setién, César Leal-Costa, María del Carmen Ortego-Mate, María Seguí-Gómez, María Jesús Durá-Ros, Mark R. Zonfrillo

**Affiliations:** 1 Nursing Department, Faculty of Nursing, University of Cantabria, Cantabria, Spain, IDIVAL Nursing Research Group; 2 Nursing Department, Faculty of Nursing, University of Murcia, Murcia, Spain; 3 Johns Hopkins Bloomberg School of Public Health, Baltimore, Maryland, United States of America; 4 Hasbro Children’s Hospital, Alpert Medical School of Brown University, Providence, Rhode Island, United States of America; Monash University, AUSTRALIA

## Abstract

**Background:**

The International Classification of Diseases (ICD) is the standard diagnostic tool for classifying and coding diseases and injuries. The Abbreviated Injury Scale (AIS) is the most widely used injury severity scoring system. Although manual coding is considered the gold standard, it is sometimes unavailable or impractical. There have been many prior attempts to develop programs for the automated conversion of ICD rubrics into AIS codes.

**Objective:**

To convert ICD, Ninth Revision, Clinical Modification (ICD-9-CM) codes into AIS 2005 (update 2008) codes via a derived map using a two-step process and, subsequently, to compare Injury Severity Score (ISS) resulting from said conversion with manually coded ISS values.

**Methods:**

A cross-sectional retrospective study was designed in which medical records at the Hospital Universitario Marqués de Valdecilla of Cantabria (HUMV) and the Complejo Hospitalario of Navarra (CHN), both in Spain, were reviewed. Coding of injuries using AIS 2005 (update 2008) version was done manually by a certified AIS specialist and ISS values were calculated. ICD-9-CM codes were automatically converted into ISS values by another certified AIS specialist in a two-step process. ISS scores obtained from manual coding were compared to those obtained through this conversion process.

**Results:**

The comparison of obtained through conversion versus manual ISS resulted in 396 concordant pairs (70.2%); the analysis of values according to ISS categories (ISS<9, ISS 9–15, ISS 16–24, ISS>24) showed 493 concordant pairs (87.4%). Regarding the criterion of “major trauma” patient (i.e., ISS> 15), 538 matching pairs (95.2%) were obtained. The conversion process resulted in underestimation of ISS in 112 cases (19.9%) and conversion was not possible in 136 cases (19%) for different reasons.

**Conclusions:**

The process used in this study has proven to be a useful tool for selecting patients who meet the ISS>15 criterion for “major trauma”. Further research is needed to improve the conversion process.

## Introduction

The Abbreviated Injury Scale (AIS), developed by the Association for the Advancement of Automotive Medicine (AAAM), is the most widely used injury severity scale in the world. It is defined as "an anatomically-based, consensus-derived, global severity scoring system that classifies each injury by body region according to its relative importance on a 6-point ordinal scale"[1]. This scale measures severity of single injuries. In order to assess the overall severity of patients with multiple injuries, the Injury Severity Score (ISS) [2] and the New Injury Severity Score (NISS) [3] were developed, based on the AIS.

The International Classification of Diseases (ICD) is also a diagnostic tool used to code and classify diseases and injuries, as well as medical procedures.

ICD was designed for administrative purposes to monitor disease trends over time and in all countries; however, it lacks specificity regarding the mechanism or the severity of injuries. The AIS, in addition to monitoring trends, was developed for researchers to encourage the development of preventive measures.

AIS and ICD are two classification systems with different lexicon and purposes. Both are relevant for injury research in order to identify the frequency and severity of injuries, monitor trends and develop prevention strategies. They are also a key element in the calculation of health costs associated with injuries, allowing hospitals the reimbursement of those costs. Injury severity information is sometimes inaccessible since it requires material and human resources that are not always available or feasible. For these reasons, several attempts have been made to create conversion programs and many have also been the difficulties encountered in the process. There are few works that compare manual coding of injuries with coding from ICD using a conversion program.

One main advantage of ICD-based scales is that information is available globally, while AIS data are only collected in some countries. European countries are in the process of incorporating AIS codes into hospital databases [4,5].

Another major drawback to the widespread use of the AIS is the need to review the entire medical record for scoring. When managing large databases for research purposes, the amount of data to be analysed makes it difficult to measure severity by manual coding.

The aforementioned limitations, have made the need to develop automated conversion programs a goal for many researchers.

The first attempt to convert ICD codes into AIS codes was carried out by Semmlow and Cone in 1976 [6].

In 1981 Gathe converted ICD, Ninth Revision, Clinical Modification (ICD-9-CM) codes into AIS (AIS 80 version) codes and on that occasion only 612 of the 2099 ICD-9-CM codes corresponding to injuries had a compatible AIS 80 score [7].

In 1988, a program called ICDMAP was created to convert ICD-9-CM codes into AIS 85 [8], and later, in 1997, into AIS 90 scores. The result was the ICDMAP-90 [9]. The automated conversion from ICD-9-CM to AIS did not always result in a one-to-one match. Nonetheless, this version of the program was validated in the paediatric population [10].

Several subsequent attempts have obtained similar conclusions regarding the problems encountered in the conversion process due to the differences between both scales. This is the case of the European Center for Injury Prevention (ECIP) whom converted the ICD-10 injury codes into AIS 98 codes [11] and Hartensuer et al. [12], who developed a methodology for the automated conversion from ICD-10 codes to AIS 2005 codes in 2015. Both reached the same conclusion, the different terminology used to describe injuries in both scales, made conversion difficult [11]. Although conversion was considered technically possible, the preliminary results questioned the quality of the conversion.

Other authors found poor concordance when comparing manual and automated conversion. This is the case of Haas et al., who created and validated an automated program to derive AIS 98 scores from ICD-10 codes [13]. Also, Di Bartolomeo et al. [14], in 2010, compared the ISS from trauma records with the ISS from automated conversion using the ICD-PIC-trauma (ICDPIC) program, freely available since 2009 to convert ICD-9-CM into AIS 98 [15]. The authors found that ISS values obtained with ICDPIC agreed poorly with manual ISS, although they concluded that conversion from ICD to AIS had great potential (14). In 2016, AAAM representatives created a program to derive AIS 2005 (update 2008) scores from ICD-9-CM and ICD-10-CM rubrics [16]. The attempt to validate the method [17] showed that the agreement between ISS calculated by expert coders and map-derived ISS values was moderate, indicating that the conversion tool was not perfect.

Recognizing the usefulness of developing a method for the ICD-to-AIS conversion, and being conscious of the difficulties found in previous attempts, the aim of this study was to convert ICD-9-CM codes into AIS 2005 (update 2008) codes via a derived map using a two-step process and, subsequently, to compare ISS resulting from said conversion with manually coded ISS values.

## Methods

A cross-sectional retrospective observational study was designed, in which medical records at the Hospital Universitario Marqués de Valdecilla of Cantabria (HUMV) and the Complejo Hospitalario of Navarra (CHN) were reviewed from February 2012 to February 2013. These are third level hospitals (equivalent to trauma centre hospitals) in two autonomous communities in the north of Spain, with similar population characteristics. The study was approved by the Clinical Research Ethics Committee of Cantabria (reference number 2015.246) All data were fully anonymized and informed consent was no required.

For sample selection, a random sampling without replacement was performed, using a computer application for the generation of random numbers in Microsoft Excel (Microsoft Corporation, Redmond, WA, USA).

Inclusion criteria for this study were: 1) patients of all ages; 2) patients who were admitted to hospital through the emergency department (inter-hospital transfers included) and discharged from the inpatient trauma service; 3) patients who were hospitalised for injuries due to external causes (ICD-9-CM codes between 800 and 959, excluding 905, 930–939 and 958, corresponding to foreign bodies, complications and consequences of injuries); and 4) for a period of one year. Exclusion criteria were: 1) patients who were not admitted through the emergency department; 2) patients who were readmitted to trauma service; 3) patients who did not present traumatic injuries; 4) patients whose medical records could not be reviewed for medical or administrative reasons; 5) patients with injuries not due to external causes; 6) patients with injuries of unknown severity (AIS = 9); and 7) cases in which conversion from ICD into ISS 2005 (update 2008) was not possible.

The sample size was calculated to ensure representativeness with the formula $n=\frac{N.Z^{2}.p(1-p)}{\left(N-1)e^{2}+Z^{2}.p(1-p\right)}$. A required sample size of 309 patients for HUMV and 342 patients for CHN was calculated based on a target population of 1116 at HUMV and of 1713 at CHN, with a 95% confidence interval, a deviation Z = 1.96, a margin of error of 5% (e = 0.05), a proportion of 50% (p = 0.5), and a possible loss of 10%. However, data from 360 and 390 patients, respectively, were collected to ensure that the required sample size was reached after exclusions.

An AIS coding specialist, certified by the AAAM and member of the International AIS Certification Board, manually coded all injuries to calculate their severity. For each case, the AIS 2005 (update 2008) code was assigned and, then, the ISS 2005 (update 2008) score was calculated.

Another AAAM-certified AIS specialist, and also member of the International AIS Certification Board, was provided with the ICD-9-CM codes of each case for conversion. ISS scores were calculated via a derived map following a two-step process. First, each ICD-9-CM code was converted to its corresponding AIS 90 code using the ICDMAP-90 software [9]. Then, those codes were manually remapped to the most recent AIS version, that is, AIS 2005 (update 2008), using the AIS manual and the ICD-9-CM injury descriptions [1,8,10]. For this purpose, a spreadsheet was developed containing the correspondence of the codes between the different versions of AIS present in the AIS dictionary. This remapping was necessary to ensure that the severity values from the most recent manual were used. Finally, ISS scores were automatically calculated from those AIS 2005 (update 2008) codes. The AIS specialist examined cases where codes could not be mapped. If the conversion was not possible, then they were excluded.

Once manual and converted ISS values were obtained, they were categorized according to the criterion described by Copes et al. in 1988 [18] as: mild (ISS between 1 and 8), moderate (ISS between 9 and 15), severe (ISS between 16 and 24), and very severe (ISS> 24). Additionally, the criterion for “major trauma”, that is, ISS> 15, was also taken into account in the current study. Although this criterion has been questioned by some authors [19–22], it is still considered the accepted definition.

For the statistical analysis, IBM SPSS version 22 program was used. As for the descriptive analysis of the sample, the mean and standard deviation of the quantitative variables and the frequency and percentage of the qualitative variables were calculated. To analyse the differences between the variables with respect to the hospital, Student’s t-tests and chi-square tests were employed according to the nature of the comparison variable.

Contingency tables were generated to compare ISS 2005 (update 2008) scores resulting from manual coding with those obtained through conversion. In addition, McNemar-Bowker χ^2^ test for polytomous categorical variables was used to test whether discordant scores (manual vs. automated) were symmetric or random in both the direction and the magnitude of disagreement.

The Cohen Quadratic Weighted Kappa Index (K) was calculated to quantify the agreement of pairs of categorized ISS values from different scoring methods (manual vs. converted), and it is a measure of the level of agreement beyond that expected by chance [23] It adjusts for chance agreement but also takes into account the degree of disagreement for tables larger than two by two by weighting the distance between pairs that are discordant. For the interpretation of the statistic κ, the classification suggested by Landis and Koch [24] was followed. According to these authors, a κ > 0.81 represents an excellent agreement; values between 0.61 and 0.80 indicate a good agreement; those between 0.41 and 0.60 represent a moderate agreement; between 0.21 and 0.40 indicate a fair agreement; values between 0.00 and 0.20 denote a slight agreement; and finally, values < 0.00 correspond to a poor agreement.

## Results

Out of the 750 patients initially reviewed, 51 were excluded: two of them because their medical records could not be accessed and the remaining 49 because they did not meet the inclusion criteria. Therefore, the final sample consisted of 699 subjects, 49.30% (n = 344) from HUMV and 50.70% (n = 355) from CHN. In the final sample, 388 were male (55.50%) and 311 female (44.40%). The mean age was 52.70 years (SD = 29.20), ranging from 0–98 years. Table 1 includes the descriptive statistics of the sample population.

**Table 1:** Descriptive analysis (sex, age and cause of injury variables) of the total sample taken by center.

*Variables*	*Total*		*HUMV*		*CHN*		
	*n = 699*		*n = 344*		*n = 355*		
	*M*	*SD*	*M*	*SD*	*M*	*SD*	*p*
Age	52.7	29.2	53.2	29.2	52.2	29.3	0.82^a^
	*n*	*%*	*n*	*%*	*n*	*%*	
Sex							
Male	388	55.5	198	57.7	190	53.5	0.21^b^
Female	311	44.4	146	42.3	165	46.5	
Causes							
Falls	422	60.4	209	60.9	213	60	
Road traffic crashes	83	11.9	52	15.1	31	8.7	
Blow, crushing and traumatic contact	112	16	46	13.3	66	18.6	
Unknown	49	7	23	6.7	26	7.3	
Assaults	16	2.3	6	1.7	10	2.8	
Self-inflicted injuries	6	0.9	3	0.9	3	0.8	0.14^b^
Exposure to hot liquids, gases or objects	3	0.4	-	-	3	0.8	
Shots and explosions	2	0.3	1	0.3	1	0.3	
Bites	2	0.3	1	0.3	1	0.3	
Respiratory obstruction	2	0.3	1	0.3	1	0.3	
Exposure to electricity, radiation and heat	2	0.3	2	0.6	-	-	

^a^t-test

^b^χ^2^ test

M: Mean; SD: Standard deviation.

In the conversion process, 135 (19.31%) cases were excluded: 73 cases (10.44%) because ICD-9 codes could not be converted into AIS 90 codes; 33 cases (4.72%) because the conversion from AIS 90 codes to AIS 2005 (update 2008) codes was not possible; 26 cases (3.72%) because the information of ICD-9 codes was poor; and 3 cases (0.43%) due to unknown injury severity.

As regards to the comparison between ISS values calculated by manual coding with those assigned through conversion, 396 concordant pairs (70.20%) were obtained, indicating good concordance. In the discordant cases, converted ISS, as compared to manual coding, resulted in underestimation of severity in 19.90% (n = 112) of the patients and in overestimation of severity in 9.90% (n = 56).

**Fig 1:**
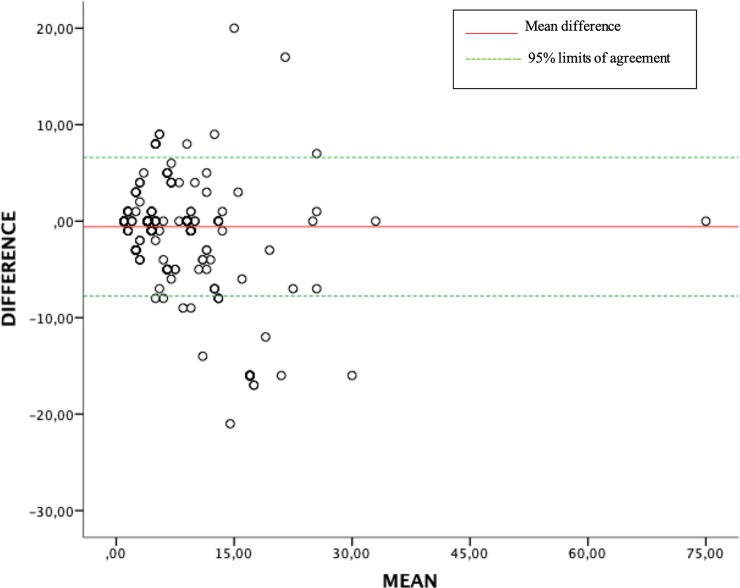
Bland–Altman plot. The difference between converted ISS with respect to manual coding is drawn against the mean of automated and manual coding in the measurements in the study.

The analysis of ISS values according to Copes´s et al. classification [18,25] showed a greater number of patients in the ISS 9–1 category for conversion, and a greater number of patients in the ISS 1–8, 16–24 and> 24 categories when coding was done manually. Thus, a total of 493 pairs (87.40%) were concordant (Table 2), obtaining a weighted Kappa index of 0.722 (p <0.05), 95% CI: 0.56–0.88, corresponding to a good agreement. The discordant cases were distributed asymmetrically around the concordant pairs (McNemar-Bowke χ^2^ = 17.79, df = 6, p = 0.007). With respect to the discordant cases converted ISS, as compared to manual coding, showed underestimation of severity in 7.97% (n = 45) of the patients and overestimation of severity in 4.60% (n = 26). We applied the original Bland–Altman method and obtained the following agreement plot (Fig 1). The mean difference (bias) of the measurements between conversion and manual coding was -0.576 and the 95% limits of agreement (-7.76, 6.60) contained 95% (535/563) of the difference scores.

**Table 2:** Comparison of ISS from manual coding versus ISS from automated conversion by categories.

		Converted ISS				Total
		Mild 1–8	Moderate 9–15	Severe 16–24	Very Severe>24	
**Manual ISS**	**Mild 1–8**	312	21	1	1	335
	**Moderate 9–15**	19	176	1	1	197
	**Severe 16–24**	1	7	1	1	10
	**Very Severe >24**	1	14	3	4	22
**Total**		333	218	6	7	564

Χ^2^ McNemar–Bowke = 17.8; df = 6; p = 0.007

K = 0.72 (p<0.05), 95% CIs (0.56–0.88)

Concordant pairs = 493 (87.4%)

Converted ISS cases lower than manual ISS cases = 45 (7.8%)

Converted ISS cases higher than manual ISS cases = 26 (4.6%)

When patients were classified according to the criterion of “major trauma” patient = ISS> 15, a total of 538 matching pairs (95.20%) were obtained. Regarding the discordant cases, with converted ISS, as opposed to manual coding, there was underestimation of severity in 4.07% (n = 23) of the patients and overestimation of severity in 0.7% (n = 4). The criterion of “major trauma” patient changed in a total of 18 patients (3.19%). Thus, with converted ISS, 2.3% (n = 13) of the patients were classified as “major trauma” (ISS> 15), whereas with manual coding, 5.67% (n = 32) of the patients were considered “major trauma” (Table 3).

**Table 3:** ISS comparison by manual coding versus ISS by mapped conversion for “Major Trauma” patient criterion ISS > 15.

		Converted ISS		Total
		Mild <15	Major Trauma >15	
**Manual ISS**	**Mild <15**	528	4	532
	**Major Trauma >15**	23	9	32
**Total**		551	13	564

Cases moved into ISS >15 by converted ISS = 4 (0.7%)

Cases moved into ISS <15 by converted ISS = 23 (4.1%)

Regarding the discrepancies in manual versus converted ISS (Table 4), the differences in the values ranged between 20 and -21 points. In 61 cases (10.80%) the difference was only of one point (1 or -1).

**Table 4:** Differences in converted ISS with respect to manual coding.

Differences converted ISS vs. manual ISS	Frequency	Percent
-21	1	0.2
-17	2	0.4
-16	12	2.1
-14	1	0.2
-12	1	0.2
-9	2	0.4
-8	5	0.9
-7	6	1.1
-6	2	0.4
-5	14	2.5
-4	7	1.2
-3	12	2.1
-2	3	0.5
-1	44	7.8
0	396	70.2
1	17	3.0
2	1	0.2
3	8	1.4
4	9	1.6
5	8	1.4
6	1	0.2
7	1	0.2
8	6	1.1
9	3	0.5
17	1	0.2
20	1	0.2
**Total**	564	100.0

Finally, the severity (ISS values based on AIS 2005 [update 2008]) obtained through manual coding of those cases in which conversion was not possible was analysed in order to know what information is missing in the conversion process. Thus, 43.2% of the cases were "mild", 48.60% were "moderate", 4.10% were "severe" and 4.10% were "very severe".

## Discussion

As said before, AIS and ICD are two classification systems with different lexicon and purposes. Both are relevant for injury research in order to identify the frequency and severity of injuries, monitor trends and develop prevention strategies. For these reasons, several attempts have been made to create conversion programs; the current study is one of such investigations.

The practicality of the tool analysed here will depend on the purpose of its use. To identify “major trauma” patients, defined as those with an ISS > 15, our results show that this system correctly reported 95.20% of the cases (it should be noted that a high percent of the sample were “minor trauma” patients). In this sense, this tool can be useful for the selection of patients according to such criterion, thus saving resources and time. It can also be especially interesting for studies with very large sample sizes, where manual coding is impractical.

However, for an accurate 1:1 conversion, the percentage of agreement decreased to 70.2%, although still acceptable and higher than that of previous studies. The more accuracy we need in estimating severity, the less reliability we obtain in the conversion process. Results of our study showed higher percentages than those of Di Bartolomeo et al. in 2010 [14] who reported a percentage of identical cases of 12.1%, and of Haas et al. in 2012 [13] who showed that coding was “similar” in 87% of the cases. According to the authors, a discrepancy of 10 points or less between manual and automated ISS is considered "similar”, which is, in our opinion, too wide a margin because 10 points on ISS could make the difference between a mild patient (ISS 1–8) and a severe patient (ISS 16–24). Our data also indicated higher percentages of agreement than those reported in more recent studies, in which concordance ranged between 48% and 54% [17]. However, the agreement between converted and manual ISS was κ = 0.722, indicating a good agreement, and the lower limit of this estimate (0.56) was comparable to other studies [13,17].

In order to determine potentially missing information, we reviewed those cases in which discrepancies in converted ISS with respect to manual ISS were found and analysed the causes of said differences. Thus, discrepancies of +/- 1 point (n = 61, 10.80%) (Table 4) were due to differences in the coding of external injuries. Usually, minor external injuries such as erosions or lacerations (AIS = 1), are not reflected in ICD coding, but they are registered by manual coding, this is why this information is lost in the conversion process, unlike manual conversion. The reasons for discrepancies of 2 to 20 points (overestimation) (n = 39, 6.90%) (Table 3) were: assignment of a higher severity code with the conversion process, differences in body regions or differences in ICD-9 coding. Finally, discrepancies of -2 to -21 points (underestimation) (n = 68, 12.05%) (Table 4) were caused by the non-identification of some injuries in the conversion process and the lack of detailed information in ICD injury descriptions.

There have been more cases of ISS underestimation (19.90%) than of ISS overestimation (9.90%) by converted coding and, moreover, the differences have been more significant. We have not found any work focused on the severity of the lost cases. The explanation can be found on the AIS itself. The golden rule of AIS coding is "code conservatively" [1]. If there is no documented information available to support the assignment of severity, the more conservative code should be chosen (i.e. a less severe AIS code in that injury´s category). This rule significantly affects the process of code conversion since ICD code information is less detailed than the information available in medical records.

As already mentioned, the severity of the cases excluded, when conversion is not possible, must be taken into account when using mapping programs, as part of the information is lost in the process. Certainly, this loss of information is the main limitation of our tool. In this study, a significant number of cases (19.46%) could not be converted. This figure is higher than in other studies [13,14] and the difference could be partly explained by the different inclusion and exclusion criteria used in those studies. Finally, some types of injuries, such as burns, drowning, asphyxia, hypothermia and frostbite, were not included in their mapping programs. However, it must be said that frequency of these injuries in our environment is very low. Di Bartolomeo et al. [14] also reported deficiencies in ICD-9 code information in their databases.

Although manual coding is still the most recommended method, when hand-coded scores are unavailable, conversion programs may be useful and practical tools, though imperfect. Overall, our study shows an underestimation of the severity of injuries when conversion is carried out, and this should be taken into account. There have been several proposals to improve these programs, such as: a) standardising injury descriptions [11,12]; b) increasing the specificity of ICD descriptions [12], c) ensuring adequate ICD information to avoid confusing information [12–14]; and d) improving mapping systems from AIS 90 and AIS 98 to AIS 2005 (update 2008) [26,27].

### Limitations

This study has some limitations. Firstly, developing double manual coding was not possible, as no other Spanish-speaking AIS coder was available when the research was conducted. So the maximum level of specialization and certification on AIS coding was required. Besides this, ICD-9 CM codes were used for the conversion, as ICD-10 had not yet been implemented in Spain at the time of data collection. Also, this conversion method tends to underestimate the severity with respect to manual coding, as well as to lose relevant information in the process. Likewise, due to the resources required for manual coding, it was not possible to analyse the entire sample, having to calculate the sample size to ensure it was representative. For this reason, the data cannot be generalized and other studies would be needed to determine the utility and reliability of the conversion tool. And finally, the vast majority of patients in the sample suffered minor or moderate injuries, so the findings of this study are not reproducible in severely injured populations.

## Conclusions

The results show that the conversion program that converts ICD codes into AIS codes performs similarly to manual coding. The observed agreement between manual and converted ISS was "good" with the chosen classification system. The kind of injured patients could facilitate those results. We have developed a method that enables the selection of “major trauma” patients, defined as those with ISS>15, in 95.2% of cases where conversion was possible. This conversion process could be useful for the identification of major trauma patients (understood as those with an ISS>15) within a certain sample or database, for research or reimbursement purposes. The authors recommend its use just when manual coding is impractical or is not available and being conscious of the information lost in the process.

Despite having obtained better results than previous studies in the 1:1 conversion, the tool needs to be improved and, therefore, changes such as the standardisation of terminology and specificity of injuries between ICD and AIS are necessary. ICD information available in databases should also be improved.

In our opinion, the key factor for the results obtained has been the participation of an AIS specialist in the conversion process.

Conversion of ICD codes into AIS codes shows a decrease, in general, in the estimation of injury severity with respect to manual coding, which should be taken into account when using this kind of tools.

The loss of information in the conversion from ICD codes into AIS codes should be quantified and assessed in order to facilitate decision-making regarding the feasibility of its use according to the objective to be attained.
